# Using mitoribosomal profiling to investigate human mitochondrial translation

**DOI:** 10.12688/wellcomeopenres.13119.2

**Published:** 2018-01-29

**Authors:** Fei Gao, Maria Wesolowska, Reuven Agami, Koos Rooijers, Fabricio Loayza-Puch, Conor Lawless, Robert N. Lightowlers, Zofia M. A. Chrzanowska-Lightowlers

**Affiliations:** 1 The Wellcome Trust Centre for Mitochondrial Research, Institute of Neuroscience, Newcastle University, Newcastle upon Tyne, UK; 2 Immunocore Ltd, Oxford, UK; 3 The Netherlands Cancer Institute, Amsterdam, Netherlands; 4 Hubrecht Institute, Utrecht, Netherlands; 5 The Wellcome Centre for Mitochondrial Research, Institute for Cell and Molecular Biosciences, Newcastle University, Newcastle upon Tyne, UK

**Keywords:** mitochondria, mitoribosomes, ribosomal profiling, human, mRNA, mitochondrial protein synthesis, codon usage, initiation

## Abstract

**Background**: Gene expression in human mitochondria has various idiosyncratic features. One of these was recently revealed as the unprecedented recruitment of a mitochondrially-encoded tRNA as a structural component of the large mitoribosomal subunit. In porcine particles this is mt-tRNA ^Phe^ whilst in humans it is mt-tRNA ^Val^. We have previously shown that when a mutation in mt-tRNA ^Val^ causes very low steady state levels, there is preferential recruitment of mt-tRNA ^Phe^. We have investigated whether this altered mitoribosome affects intra-organellar protein synthesis.

**Methods**: By using mitoribosomal profiling we have revealed aspects of mitoribosome behaviour with its template mt-mRNA under both normal conditions as well as those where the mitoribosome has incorporated mt-tRNA ^Phe^.

**Results**: Analysis of the mitoribosome residency on transcripts under control conditions reveals that although mitochondria employ only 22 mt-tRNAs for protein synthesis, the use of non-canonical wobble base pairs at codon position 3 does not cause any measurable difference in mitoribosome occupancy irrespective of the codon. Comparison of the profile of aberrant mt-tRNA ^Phe^ containing mitoribosomes with those of controls that integrate mt-tRNA ^Val^ revealed that the impaired translation seen in the latter was not due to stalling on triplets encoding either of these amino acids. The alterations in mitoribosome interactions with start codons was not directly attributable to the either the use of non-cognate initiation codons or the presence or absence of 5’ leader sequences, except in the two bicistronic RNA units, *RNA7* and *RNA14* where the initiation sites are internal.

**Conclusions**: These data report the power of mitoribosomal profiling in helping to understand the subtleties of mammalian mitochondrial protein synthesis. Analysis of profiles from the mutant mt-tRNA ^Val^ cell line suggest that despite mt-tRNA ^Phe^ being preferred in the porcine mitoribosome, its integration into the human counterpart results in a suboptimal structure that modifies its interaction with mt-mRNAs.

## Introduction

Understanding the process of protein synthesis and determining the features that govern the progression of the ribosome along its template RNA have been areas of interest for many years. A recent wave of publications to address these long standing questions was initiated by the technical development of ribosome profiling by Ingolia and colleagues ^1^. The approach uses high density sequencing of ribosome-protected mRNA fragments to identify the positions within the transcript where there is an accumulation of ribosomes. There are many features that can contribute to the pausing and processivity of the synthetic machinery as it translates its template mRNA and it is becoming apparent that this is not as simple as, or restricted to, the secondary structure of the RNA ahead of the ribosome. Pausing may also be regulated by the abundance of charged tRNA, the number of consecutive codons for the same amino acid ^2^, the charge of the newly synthesised peptide, folding and secondary structure of the peptide fragment within the exit tunnel ^3, 4^, the influence of non-canonical wobble bases in codon:anticodon pairing ^5^ or by chemical damage of mRNAs or their degradation such that they lack a stop codon. Transient accumulation of ribosomes can also be observed at sites of translation initiation, whilst ribosomes are localising to particular subcellular locations ^6^ or during recruitment of chaperones or regulatory factors ^7^.

To date, much of the evaluation of ribosomal profiles has been qualitative ^8^, which has provided useful and interesting insights. As a result, new quantitative algorithms have been developed, which have improved the quantitative interpretation of the large data sets that are generated by the high-throughput sequencing. Quantitative profile evaluation allows sensitive statistical analyses that would not be achievable through the old style cloning and sequencing ^9– 11^. Ribosome profiling has been applied to a number of systems including *Caulobacter*, *Plasmodium*, yeast, zebrafish, rat and *Arabidopsis* as well determining changes in response to different physiological conditions ^12– 18^. A number of studies have looked at the ribosome profiles of nuclear encoded transcripts of mitochondrially-destined proteins, under different conditions or in the proximity of mitochondria compared to other subcellular compartments ^16, 19– 21^ but there has been less interrogation of the ribosome profile of the human mitochondrial transcriptome ^22^.

The first publication describing ribosomal profiling in human mitochondria focussed on the consequences of a mutation in *MT-TW*, the gene encoding mitochondrial tRNA tryptophan ^22^. The data from that study saw an increased protection of many but not all triplets corresponding to tryptophan and a reduction of the proportion of protected RNA fragments downstream, which was interpreted as a change in translation rate due to the altered progression through the remainder of the open reading frame ^22^. The scope of this elegant study restricted itself to the consequences of the mutated tRNA participating in protein synthesis, leaving many of the actual features associated with mitoribosome profiles unanalysed under control conditions. Mitoribosome distribution has also been assessed following administration of an arginine analogue to determine the effect of this misincorporation on intra-organellar protein synthesis ^23^. These data limited the comparison of mitoribosome distribution to only *MTCO1* transcript with and without treatment, and the frequency of arginine codons found within protected footprints compared to untreated controls. Since there are many ways to analyse the output, the data we present here, although not comprehensive, provides a broad analysis, under control conditions, of features relating to the coding triplets found within protected fragments and also across open reading frames.

It is now known that the mitochondrially-encoded tRNA ^Val^ is incorporated as a structural component of the human mitoribosome ^24– 26^. This unprecedented incorporation of a tRNA rather than a 5S rRNA is also seen in other mammalian mitoribosomes but appears to be restricted to either mt-tRNA ^Phe^ or mt-tRNA ^Val^ ^24, 27^. We recently published that in human cells with a mutated, unstable mt-tRNA ^Val^ there is a preferential incorporation of mt-tRNA ^Phe^ into the mitoribosome ^27, 28^. Since this mt-tRNA is found in porcine and bovine mitoribosomes, it is possible that this substitution would generate fully functional mitoribosomes. Here we present data concerning mitoribosome pausing not only from control cells but also from our extended investigations determining whether substitution of mt-tRNA ^Val^ for mt-tRNA ^Phe^ as the structural mt-tRNA incorporated into mt-LSU ^27^ has an effect on these profiles.

## Methods

### Cell culture

Human 143B.206 Rho ^+^ cells and cybrid derivatives ^27^ were cultured (37°C, humidified 5% CO _2_) in DMEM (Sigma) supplemented with 10% (v/v) foetal calf serum, 1x non-essential amino acids, 50µg/mL uridine, and 2mM L-glutamine.

### Generation and analysis of mitoribosome protected fragments

These were generated as described in Rooijers *et al.* ^22^. Control ribosome profiling data are deposited in NCBI Gene Expression Omnibus (accession codes GSE48933). Raw data from experimental samples is deposited in ebi ArrayExpress (E-MTAB-6284). Libraries were sequenced by Edinburgh Genomics, Genome Science on a high throughput Illumina HiSeq2500 platform. Adapter sequences were removed using cutadapt (version 1.4.2) and the trimmed fastq files were filtered using bowtie2 (version 2.2.3) to remove tRNA and rRNA sequences from both nuclear and mitochondrial genomes as described in 22. The remaining sequences were then aligned to the human mitochondrial reference sequence using bowtie2 ^22^.

### Identification of mitoribosome pausing

To facilitate direct comparison between profiles we generated fractional profiles. Footprint fractional profiles were generated by dividing footprint profiles by total number of reads per replicate. A fractional abundance profile was generated by dividing the mtDNA abundance profile by the total number of codons within the coding region of mtDNA, where the total of all signals was equal to 1.

To identify motifs associated with mitoribosome pausing, we carried out multiple, one-sample, two-tailed t-tests comparing three replicate observations of the fractional footprint profile values with fractional abundance profile values at each codon using the t.test function in R (version 3.4.1). This procedure generated a list of p-values for the statistical significance of differences observed at each codon. In order to account for the Type I error associated with multiple testing of 64 codons, we used FDR correction to generate a list of corrected q-values (using the p.adjust function in R). We classified differences where q < 0.05 as significant. We repeated this procedure for the wild-type cell line and the mutant cell line separately.

To identify the effects of preferential integration of mt-tRNA ^Phe^, we carried out multiple, two-sample, two-tailed t-tests comparing three replicate observations of the fractional footprint profile values from the wild-type cell line with three replicate observations of the fractional footprint profile values from the mutant cell line. As above, we first generated 64 p-values and corrected for multiple testing to give 64 corrected q-values, classifying differences where q < 0.05 as significant.

### Plots of footprints covering the 5’ termini

All footprints that started from the very 5’ terminus to 5 nucleotides downstream of the of the start codon, were mapped to their nucleotide position of the mtDNA reference sequence ^29^, irrespective of their individual lengths (x-axis represents the nucleotide position in the reference mtDNA). These footprints were mapped as blocks where the depth (y axis) of the block is proportional to the frequency with which that specific length of footprint, in that position, is found as part of the selected sequences from the library.

### Profile clustering

We generated tRNA footprint profiles from sequencing data by summing up the number of reads at each codon in the coding region of mtDNA. We generated an mtDNA abundance profile by counting the number of occurrences of each codon within the coding region of mtDNA. Differences between tRNA footprint profiles for the three replicates from the wild-type strain, the three replicates from the mutant that preferentially integrates mt-tRNA ^Phe^ and the mtDNA abundance profile were examined by unsupervised hierarchical clustering. Differences between profiles *i* and *j* were defined as *1 - P _ij_*, where *P _ij_* is the Pearson's correlation coefficient between profiles *i* and *j*. Clustering was carried out using the hclust function in the statistical programming language R and results were visualised using the corrplot function and hclust functions in R.

## Results

Since little has been published thus far on the functional characteristics of human mitoribosomes, we have defined a number of questions to explore patterns of behaviour. We have then compared the behaviour of the control human mitoribosome, which has a mt-tRNA ^Val^ component, against that of the cell line that has preferentially incorporated mt-tRNA ^Phe^, as described above, in order to highlight the power of mitoribosomal profiling for detecting subtle alterations in ribosome function.

### Analysis of the codon coverage within the protected mitoribosome footprints

The first question we addressed was whether the frequency with which each coding triplet is found within the mitoribosome protected footprints, directly reflected how often the triplet is found within the coding sequence of human mtDNA. One potentially confounding issue is the use of wobble base pairs. The human mitochondrial genome encodes only 22 tRNAs. This necessitates the use of wobble base pairings to permit the entire set of codon:anticodon interactions ^30^. Since the use of a wobble represents an imperfect codon;anticodon it was possible that such mismatches could affect the dwell time of some aminoacylated mt-tRNA within the mitoribosome. If the dwell time is increased for inexact versus exact codon pairings, the mitoribosome residency would be increased and therefore the fractional abundance for those codons within protected fragments would be higher than would be predicted from the calculated fraction of the same imperfect codons in the thirteen ORFs encoded in the mitochondrial genome.

**Figure 1.**
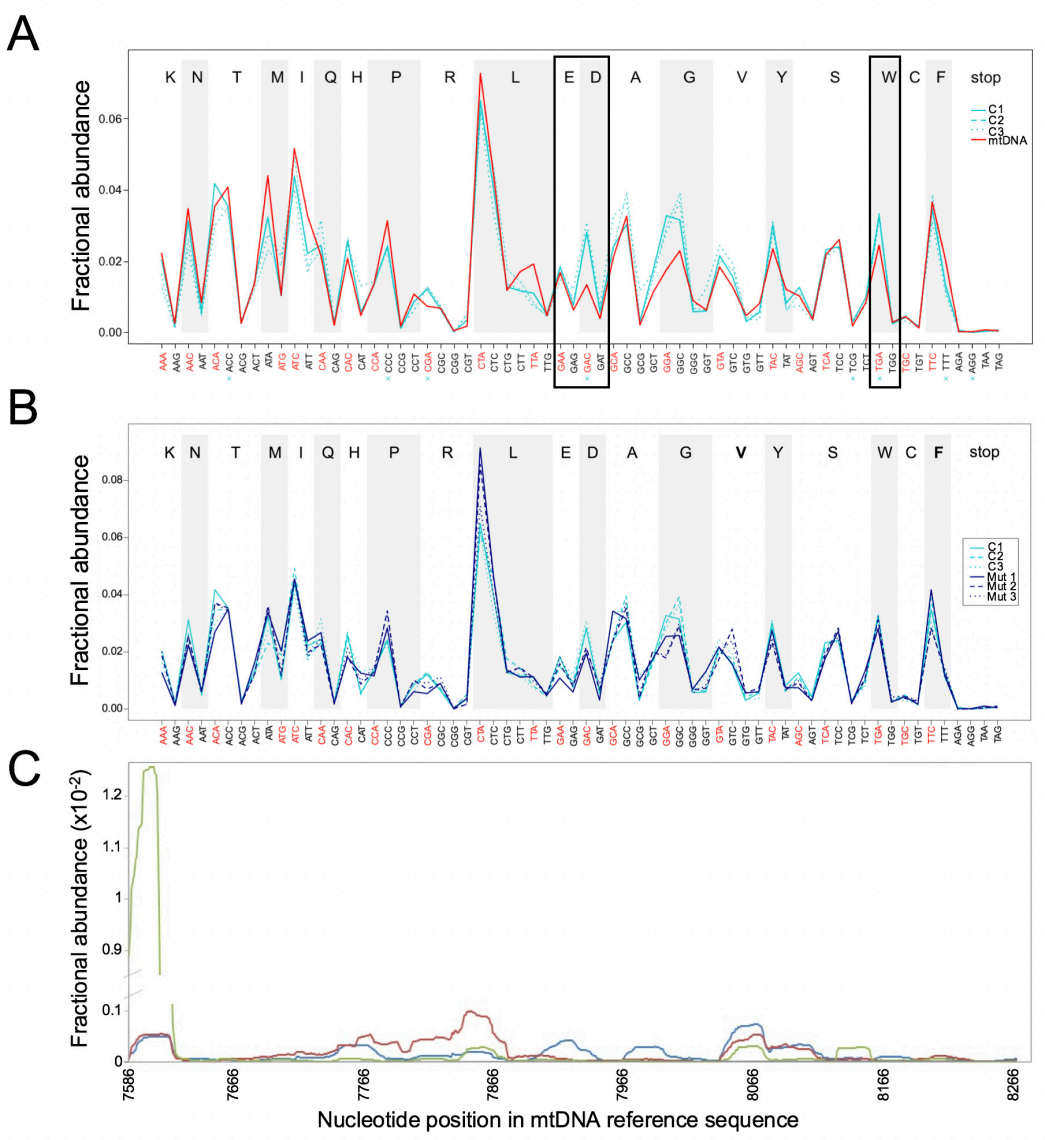
Mitochondrial codon usage in control and mutant mitoribosome. Mitoribosome footprints were analysed for codon coverage and their abundance compared to codon usage in the human mitochondrial genome. The frequency with which each of the 64 codons is present in the open reading frames of human mtDNA was calculated (mtDNA). This was then compared to the proportion of which each codon was found in the total of all protected RNA fragments represented in the libraries generated from the three control cell lines (C1, 2, 3) ( **A**). The data presented is FDR corrected, to generate a list of corrected q-values (where q < 0.05 is regarded as significant and marked by a blue cross beneath the codon). The boxed areas represent regions described in main text. **B**. The same analysis of mitoribosome coverage of each codon was performed comparing the control (C1-3; n=3) with the mutant cell line (M1- 3; n=3), which had substituted mt-tRNA ^Val^ with the mt-tRNA ^Phe^. **C**. The mitoribosome distribution across *MTCO2* for 3 different cell lines was plotted as a proportion of the footprints found over all the mt-ORFs in the library (fractional abundance). 143B.206 – blue, 143B cybrid – red, HEK293 – green.

The frequency of each codon present in the 13 open reading frames (ORFs) of human mtDNA was therefore plotted as the proportion (fractional abundance) found within the total reads from our protected RNA fragment libraries of mtDNA ORFs. Libraries derived from control osteosarcoma 143B.206 parental cells were generated from biological triplicate experiments ( Figure 1A; Control, C; three biological repeats in cyan; mtDNA in red) and showed good reproducibility of data ( Figure S1). The fractional abundance largely follows the frequency of the coding sequence with a number of exceptions where there is a statistically significant difference (marked by a blue cross below the codon). These differences do not occur for any set of codons representing a specific amino acid, nor are they restricted to wobble bases (designated in black text), suggesting that imperfect base pairing is not an impediment to translational efficiency ( Figure 1A, perfect codon:anticodon pairs labelled in red text). For example, we find no evidence that the fractional abundance of either GAA or GAG (glutamic acid) codons in our protected RNA fragments differ significantly from their abundance within the sequence of the mtDNA-encoded open reading frames despite GAA generating a perfect codon:anticodon pair, and GAG requiring a wobble. The aspartic acid GAC codon generates a perfect codon:anticodon pair whilst the GAU codon does not. In contrast, this canonically matched pairing displays significantly higher mitoribosome residency than the fractional abundance in the ORF sequences. Further, in human mitochondria UGA is a codon that has been recoded from a canonical stop to be recognised by mt-tRNA ^Trp^ and although the codon:anticodon pair is a perfect match, the mitoribosome residency is significantly greater than expected when compared to the fractional abundance in the mtDNA sequence. This is in contrast to the alternative tryptophan UGG codon that forms an imperfect pair, but where we found no difference to the fractional abundance predicted by the mtDNA ORFs.

Our previous analysis of a cell line harbouring a mutation in mt-tRNA ^Val^ identified a reduction in the steady state levels of mt-tRNA ^Val^ and that the presence of this destabilising mutation results in the substitution of mt-tRNA ^Phe^ into the large mitoribosomal subunit ^27, 28^. Although one might predict that this indicates an interchangeable arrangement that should not affect function, metabolic labelling of this cell line suggested that intramitochondrial protein synthesis was subtly impaired. The assumption at the time was that this was due to the reduced availability of charged mt-tRNA ^Val^ ^28^. Further, since this cell line has integrated a fraction of the mt-tRNA ^Phe^ into the mitoribosome, there was a decreased proportion available for incorporation into elongating peptides. One hypothesis would be that mitoribosomes would pause over phenylalanine codons as there would be an extended time before pairing with an appropriately charged mt-tRNA ^Phe^ could occur. This premise would predict a greater relative abundance of phenylalanine codons in the resultant library of protected RNA fragments. We, therefore, aimed to identify whether the substitution of the structural mt-tRNA in the mitoribosome caused any pausing over the codons encoding either of these amino acids. Profiling data were collected from three biological repeats of the control (C; containing mt-tRNA ^Val^) and mutant (Mut; containing mt-tRNA ^Phe^) cell lines ( Figure 1B). Surprisingly, no statistically significant differences in mitoribosome accumulation were identified over any of the codons. Most relevant, this was also true for all of the Val or Phe coding triplets, where there was no evidence of mitoribosome accumulation despite the lowered steady state levels of mt-tRNA ^Val^, and the depletion of available charged mt-tRNA ^Phe^ due to its incorporation into the large subunits. This suggested that availability of each of these mt-tRNAs was not limiting peptide elongation during the translation process.

Although this provides insight into codon usage it does not address the coverage or representation on individual transcripts, which requires the data to be interrogated in a different way.

### How are the mitoribosomes distributed within open reading frames?

Data from Agami and colleagues described two sizes of mitoribosomal footprints with similar distribution across the transcripts analysed. They hypothesised that this reflected the altered susceptibility of mt-mRNAs to RNases, due to different conformations adopted by the mitoribosome during translation elongation ^22^. Our data was consistent with footprint sizes spanning 24 to 37 nucleotides. By plotting the distribution of these mitoribosomal footprints as a proportion of the total that are found across all the individual ORFs, the pattern of mitoribosome accumulation across the transcript can be easily viewed, as typified by the profile on *MTCO2* ( Figure 1C, and *MTCO1* in Figure S1C). A similar trend but not identical trace was observed when different cell lines were analysed, including 143B osteosarcoma, *trans*mitochondrial cybrid and HEK293 cell lines. Subsequent interrogation of the codons in the footprint, as well as the sequences flanking it may provide an explanation for any observed accumulation.

Previous *in vitro* studies by Spremulli and colleagues investigated the interaction of the 55S monosome and the 28S mt-SSU with RNA templates that had AUG start codons with differing numbers of nucleotides upstream ^31^. They were able to show that the mitoribosome binds the mt-mRNAs with a strong preference for start codons at the very 5’-terminus. Although eight of the human mt-mRNA species have the initiating codon in this position, this lack of untranslated regions (UTRs) is not universal. In fact, only 4 of the 13 open reading frames lack both associated 5’ and 3’ UTRs. Two transcription units are bicistronic with overlapping reading frames. One of these units, *RNA14,* encodes two components of the F _o_F _1_ ATP synthase, complex V of the OXPHOS system. The distal *MTATP6* element is out of frame with the ORF for ATP8 and essentially acts as a 3’UTR for the upstream element. This arrangement also means that the 5’ proximal *MTATP8* coding sequence acts as the 5’UTR for *MTATP6*. A similar overlapping arrangement is found in *RNA7* that encodes 2 subunits of complex 1, ND4 and ND4L. Three ORFs have leaders of 3 nucleotides or fewer ^32^, one of which is *MTCO1* with 3 nucleotides ahead of the start codon. The first 151 nucleotides of this transcript was used as a template by Spremulli and colleagues. Their analyses showed that the addition of even a single nucleotide ahead of the start codon for *MTCO1* reduced binding, and 3 nucleotides decreased formation of an initiation complex by more than 40% ^31^. We, therefore, aimed to identify if the absence or presence of nucleotides preceding the initiating codon affected the mitoribosome residency over the initiation site in these transcripts in mitoribosome profiles from live cells. Further, since human mitochondria use AUA and AUU in addition to AUG as start codons, we also analysed whether the mitoribosome residency was affected by the non-canonical start codons. Interestingly, all the unconventional start codons are found in transcripts that have no 5’ UTRs, so that the first 5’ nucleotide in the transcript corresponds to the first A residue of the initiation codon.

**Figure 2.**
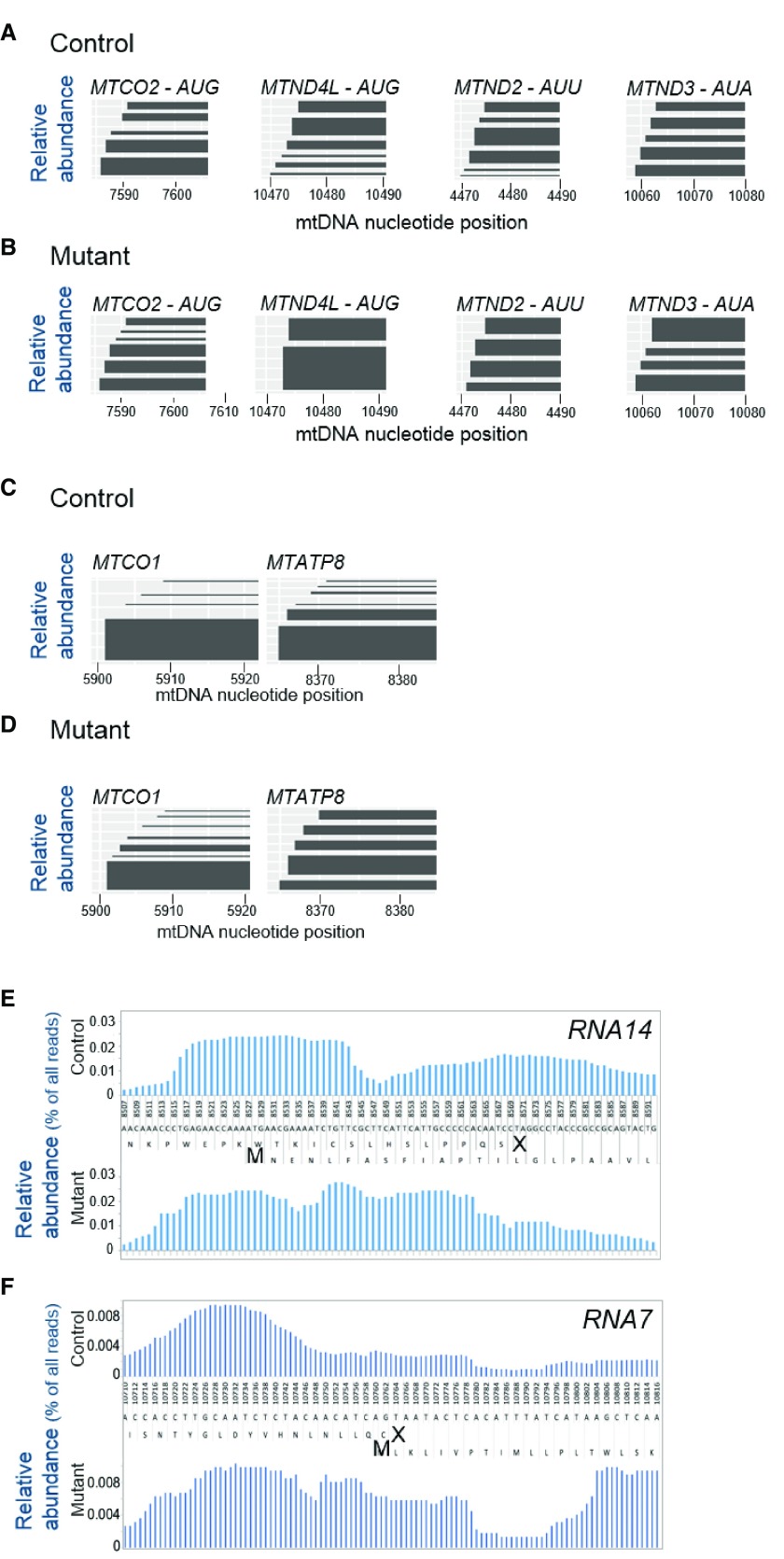
Mitoribosomal footprint distribution at the 5’ terminus of mt-transcripts Species that protected the 5’ terminal nucleotide of the transcript, or that mapped within 5 nucleotides downstream of the initiator codon were analysed as a pool and those that initiated with the same initial 5’ nucleotide were grouped together. The first 20 nucleotides of each group is plotted against the mtDNA reference sequence (x-axis) and the relative abundance is represented by the depth of each footprint block (y axis) calculated as a proportion of the population of protected species found at the 5’ terminus). Four transcripts that have start codons at the very 5’ terminus were analysed and the footprint position, length and relative abundance compared between control ( **A**) and substituted mitoribosomes ( **B**, Mutant). Mitoribosomal footprints on transcripts *MTCO1* and *MTATP8* with 3 or 1 nucleotides respectively that precede the start codon were mapped as above, for the control ( **C**) and substituted mitoribosomes ( **D**, Mutant). Panel ( **E**) shows the raw IGV data of the mitoribosome footprints from the region of the bicistronic transcript, *RNA14*, where the reading frames of the *MTATP8* and *MTATP6* genes overlap. The control data is above and mutant data below. The nucleotide positions from the mtDNA reference sequence are in between with the two translation frames. The upper amino acid sequence relates to ATP8 with the termination site indicated by X. The lower sequence corresponds to the downstream ATP6 with the initiating methionine (M) shown in bold. Panel ( **F**) depicts the overlap of *MTND4L/MTND4* in *RNA7* a similar way.

First, we found a substantial number of protected fragments that contained a 5’ residue corresponding to the initiation codon at, or very near to, the 5’ terminus of mature transcripts (e.g. Figure 1C, Figure S1). This was a surprise as these fragments ranged from 24–37 nucleotides and few were sufficiently small (15-17nt) to have mapped their initiation codon at the P site of the mitoribosome. Attempts to enrich other libraries for shorter protected species were unable to identify significant numbers of such short fragments that protected the initiation codon. We are not currently in position to interpret this data, but it is intriguing to speculate that it may indicate that protected fragments from preinitiation complexes are rare. We, therefore, analysed the population of footprints that covered the 5’ terminal nucleotide of the transcript, or that initiated within 5 nucleotides downstream of the start codon. From within this population, all protected sequences that started with the same initial 5’ nucleotide were grouped together. These groups were aligned against their nucleotide position in the reference mtDNA sequence ( Figure 2A, x axis). The relative abundance (y axis) of each of these groups (protected fragments with the same 5’ start site) is represented by the depth of each footprint block and is calculated as a proportion of footprints within that population of protected species found at the 5’ terminus. Two of the open reading frames that lack 5’ UTRs are *MTCO2* and *MTND4L* ( Figure 2A). In each case AUG is the initiating codon. Protected sequences in the former, indicate that 39% include the 5’ AUG (bottom block) after which the mitoribosome footprints shift into the open reading frame with a smaller proportional abundance at each site. This differs from the pattern seen for *MTND4L*, where only 5.4% of protected sequences cover the AUG start codon. The mitoribosome coverage at the 5’ terminus for *MTND2* was also low at 2.8% despite this transcript initiating translation from the unconventional AUU. *MTND3* also uses a recoded start codon, in this case AUA, but here 22.8% of the footprints cover the 5’ terminus, with a more equal distribution in each position as the mitoribosome moves into the open reading frame. This pattern more closely resembled that seen in *MTCO1*, which uses the conventional AUG start codon.

These data suggested that the unconventional start codons do not have a strong impact on mitoribosome occupancy under control conditions, but do they alter the interactions with a mitoribosome that had integrated mt-tRNA ^Phe^ instead of mt-tRNA ^Val^ as the structural component of the mt-LSU? For the *MTCO2* transcript, the substituted mitoribosome distribution was very similar to control. The pattern of *MTND4L* in control showed a small proportion of the footprints at the very 5’ terminus with the distribution of the remaining footprints commencing over the next 5 nucleotides. The distribution of footprints from the mutant mitoribosome differed as the vast majority of protected species commenced 3–4 nucleotides after the start codon ( Figure 2B). Since both *MTND4L* and *MTCO2* use a canonical AUG as a start codon, and only the pattern for *MTND4L* is altered in the substituted mitoribosome, the differences in the footprint distribution are unlikely to be related to the ability of the substituted mitoribosome to recognise AUG. There were more modest differences in mitoribosome behaviour between the control and mutant over both the AUA codon of *MTND3* and the AUU of *MTND2*. In each case the footprints from the mutant mitoribosome were staggered over four positions compared to a slightly different relative abundance distributed across the five footprints in the control ( Figure 2B).

Two of the transcripts with limited non-coding sequence at the 5’ terminus are *MTCO1* and *MTATP8*, with 3 or 1 nucleotides, respectively, upstream of the initiating codon. Inspection of the control mitoribosome footprints at the 5’-terminus of these two transcripts reveals a distribution that is in stark contrast to those seen on the four transcripts described above that lack non-coding sequence at the 5’ terminus. Here, the footprints on *MTCO1* and *ATP8* showed a similar distribution with the majority of protected sequences, 86% and 62% respectively, covering the non-coding nucleotides ( Figure 2C). This difference in mitoribosome behaviour is consistent with the *in vitro* observation that initiation is affected by nucleotides upstream of the start codon ^31^. Do these minimal 5’UTRs have an even greater impact on the interaction with aberrant mitoribosomes containing mt-tRNA ^Phe^? For *MTCO1* the majority, albeit reduced (64% *cf* control 86%) of the footprints were still found to be covering the non-coding nucleotides after which the mitoribosome footprints moved downstream in a more incremental fashion than seen in the control ( Figure 2 panels C and D). The redistribution of the mutant mitoribosome footprints on the transcript encoding ATP8 was more distinct. Whether it was the presence of a single nucleotide 5’ proximal to the initiating AUA or the use of an unconventional start codon, the consequence was a redistribution of the mutant mitoribosome footprints. Whilst in the control the majority (62%) of footprints were positioned over the nucleotide preceding AUA, this diminished to 18% with the remainder of footprints being more evenly distributed across largely the same positions as found in the control ( Figure 2 panels C and D).

The *MTATP6* initiation codon is embedded within the bicistronic *RNA14*, such that the upstream out-of-frame ATP8 coding region acts as a 5’UTR. The start codon for translation of ATP6 is a canonical AUG, however, due to its internal position, the mechanism by which the mitoribosome recognises this initiation site remains unclear. Since there are 162 nucleotides preceding the start site, this region may act as an internal ribosomal entry site (IRES) to recruit mitoribosomes. Examining the pattern of mitoribosome residency reveals that the control mitoribosome generates distinct footprints centred over the ATP6 initiation site ( Figure 2E, upper panel). Immediately downstream there is a second footprint with a lower relative abundance of protected fragments that spans the stop codon for ATP8, which is displaced by 40 nucleotides from the ATP6 initiation site. In contrast, the substituted mitoribosome containing the mt-tRNA ^Phe^ recognises neither the internal start codon nor the stop codon with the same precision as the control ( Figure 2E, mutant lower panel). For *RNA7*, the other bicistronic RNA unit, the overlap of coding sequence is limited to a single nucleotide intervening between the AUG start codon of *MTND4* and the UAA stop of *MTND4L*, compared to 40 nucleotides separating the start and stop codons in *RNA14*. Again, the mechanism of mitoribosome recruitment to this site is undefined and this region may also act as an IRES. The overlap, however, is restricted to so few nucleotides that a -1 frameshift, which human mitoribosome have been shown to perform ^33^, would position the AUG in the P-site to facilitate initiation. Analysis of this region showed that control cells displayed a distinct mitoribosome accumulation upstream of the sequence spanning the AUGCUAA overlap, in a region predicted to be an open loop, followed by a lesser accumulation over the stop/start transition predicted to fall within a short stem ( Figure 2F, upper panel, Figure S2). The distribution of the substituted mitoribosome differs from this, with an increased residency slightly further downstream and a change in the proportion of protected footprints just ahead of and over the stop/start transition compared to control. This profile displays an increase in the relative abundance of mitoribosomes where the open reading frames overlap ( Figure 2F, lower panel). The substituted mitoribosome behaviour at stop sites was not always altered, even when the termination codon was followed by a short non-coding stretch of nucleotides, as the traces around the stop codon of *MTCO2* were almost identical ( Figure S3). This change in profile, together with that seen for *RNA14* suggests that the substituted mitoribosome has more difficulty acting at these transition sites, and that despite the use of a conventional AUG start codon, the initiation at putative internal entry sites appears to be disrupted.

Our data indicate that there is no facile relationship between position or sequence of initiation codon and the relative occupancy of the mitoribosome at either the 5’ termini, or embedded start sites of these transcripts. RNA structures downstream of the footprint, however, might be expected to contribute to the processivity of the mitoribosome. Prediction programmes for RNA structures have been refined significantly but without experimental confirmation they remain provisional. To overcome this, the Spremulli group used SHAPE chemistry to analyse the interaction of the mitoribosome with the mt-mRNAs ^34^. This allowed them to determine the structures and respective stability of the 5’-termini of various human mt-transcripts, which were different to those predicted by RNAstructure ^35^. The substituted mitoribosome behaved differently when initiating translation on a number of ORFs including all four encoded on the two bicistronic elements ( *RNA7* and *RNA14*). The differences in behaviour were seen both at the 5’-termini as well as at the internal start sites of both downstream ORFs. SHAPE chemistry determined that the 5’-termini of both *MTND4L* and *MTATP8* have the ability to generate single stem loops. In the case of *MTND4L* the initiating AUG is found as the first nucleotides in the paired stem, whilst for *MTATP8* this structure is 1 nucleotide downstream of the start codon. Although the footprint profiles over or close to the start codon might suggest that the recognition and effective interaction with the mutant translation machinery is compromised, neither of these structures is predicted to be more stable than a single G-C base pair ^34^ and so is unlikely to impede progress of the mitoribosome. The internal initiation codons in both *RNA14* and *RNA7* were determined to be highly unstructured regions ^34^. Hence, RNA structure was unlikely to be the cause of the difference in the recognition of the signals and behaviour of the mutant mitoribosome in the regions encompassing the start/stop transitions. The *MTCO1* transcript also showed a difference in behaviour at the initiation codon in the control and mutant cell lines. In addition to the effect of nucleotides preceding the start codon, SHAPE chemistry determined the AUG start to be in a conformationally flexible region with the first reasonably stable secondary structure to be 39 nucleotides downstream ^34^. It is possible that the first stem loop that occurs just ahead of the mitoribosome after it has initiated translation, selectively affects the progress of the mt-tRNA ^Phe^ containing mitoribosome compared to the control.

It has been proposed that in addition to the influence of the mRNA sequence or structure, the characteristics of the translation product itself may influence the local interactions and alter the transit time of the nascent peptide through the ribosomal exit tunnel, thus restricting the movement of a ribosome on its template mRNA ^25^. The human mitoribosome appears to have adapted the exit tunnel to accommodate for the highly hydrophobic nature of the nascent peptides. CryoEM characterisation of the mt-LSU shows particular involvement of uL22 in facilitating hydrophobic interactions that could enhance interactions of the translation product with the tunnel wall ^25^. It also indicates that the stretch of newly synthesised protein closest to the exit site can have already adopted a helical formation ^25^. Any condensation of the structure would also make it difficult to determine the overall length of each translation product within the tunnel once the mitoribosome had progressed along the transcript. To determine whether the characteristics of the initial peptide could explain the mitoribosome profiles we inspected the hydropathicity predictions of the initial 10 amino acids of transcripts described above. Using ExPASy programmes where a positive score indicates hydrophobicity ^36, 37^, these products were predicted to generate hydrophobic peptides. The most hydrophobic was the translation product of *MTND4* with a hydrophobicity score of 1.84, followed by *MTND3* (1.80), then *MTCO2* and *MTND2* both with a score 0.47. Since the substitution of mt-tRNA ^Val^ for mt-tRNA ^Phe^ may have structural implications for the mitoribosome, which we discuss later, we wished to identify if the difference in behaviour we observed at early stages of translation correlated with the hydrophobicity prediction. Of these eight transcripts analysed, including both elements of the two bicistronic elements, six showed footprints that differed from the control. The altered footprint distribution did not, however, reflect the hydrophobicity of the initial peptides, as the most distinct differences in mitoribosome behaviour were seen on the transcripts for *MTND4L* and *MTATP8* with widely different hydropathicity predictions of 1.59 and -0.26 respectively. The differences in this characteristic do not easily explain the mitoribosome residency patterns seen around this initiation sites of these transcripts. Since the hydrophobicity did not appear to exert an effect, we looked at the structural prediction for the initial peptide sequence ( Table S1). Of the analysed transcripts only the translation product of *MTND2* showed any helical propensity, suggesting that the formation of a structure within the exit tunnel was not a major factor in mitoribosome residency during the initiation stages for these transcripts. We could not, therefore, attribute any changes in relative translational efficiency of the mt-tRNA ^Phe^ containing mitoribosome to either the hydrophobic character or the structure of the initial peptide.

## Discussion

Our analyses reveal that a human mitoribosome that substitutes mt-tRNA ^Phe^ into the large subunit as a structural component in preference to a mutant mt-tRNA ^Val^, displays subtle differences in its interaction with mt-mRNA templates when compared to the control. We have examined a number of elements that might have been predicted to contribute to this change in behaviour but have not found any convincing evidence to support a simple explanation.

Unfortunately, there is currently insufficient detailed structural data on how either the human mt-tRNA ^Val^ or mt-tRNA ^Phe^ are held within the mt-LSU to be able to predict precisely how insertion of the latter might affect the conformation of the mt-LSU in the context of the monosome, to drive these differences in behaviour. Nor do we currently know the extent to which either mt-tRNA ^Phe^ and mt-tRNA ^Val^ are modified when they are integrated into the mt-LSU. Thus, we cannot determine whether differences in post-transcriptional modifications change the charge distribution or alter the conformation of the monosome, particularly with respect to the central protuberance (CP). This is important, as despite the exchange of a 5S rRNA for a mitochondrially encoded tRNA the central protuberance has retained two functions in common with its bacterial 70S counterpart. Unlike the 70S, these functions involve mitochondrial specific proteins that facilitate the i) interaction of the mt-LSU with the small subunit and ii) interaction with the mt-tRNAs. Although the cryoEM data cannot distinguish the precise elements that are involved in mediating these functions it can assign a mitochondrial specific mass at the base of the central protuberance, which crucially is coordinated by mt-tRNA ^Val^ ^25^. These proteins are clustered in a region that is predicted to undergo structural rearrangement during translation ^38^ and the flexibility to alter the conformation is believed to be expedited by mt-tRNA ^Val^ ^25^. Thus, the substitution of mt-tRNA ^Val^ for mt-tRNA ^Phe^ may have functional consequences on the central protuberance by affecting the potential of the mt-LSU to change in flexibility/conformation and may in part be the cause of the altered behaviour of the substituted mitoribosome.

We hope at some stage in the future it will be possible to generate cryoEM structures from the mt-tRNA ^Phe^ containing mitoribosome to gain insight into how a structural change may have contributed to this alteration in translation. Such structures may reveal changes in conformation following mt-LSU incorporation of the mt-tRNA ^Phe^ that indicate whether it is the fidelity of translation that is reduced, causing the production of aberrant polypeptides that are more rapidly degraded. This would explain the lowered steady state levels of mtDNA encoded proteins previously observed in cell lines harbouring the mt-tRNA ^Val^ mutation ^28^.

A mitoribosome profiling approach could be also used to reveal the pathological mechanisms underlying many other mutations that affect mitochondrial gene expression at a molecular level. In addition to the example we have presented, mitoribosomes have been identified as accumulating over the different codons encoding tryptophan as a consequence of a mutation in the mitochondrially encoded tRNA ^Tryp^ ^22^. It is probable that mutations in other mtDNA encoded tRNAs or in the enzymes responsible for their maturation and modification may also affect codon usage. Further, it would be interesting to identify whether any changes in codon usage from such mutations can be correlated to the frequency of a particular codon in the genes of different OXPHOS complexes, thereby explaining the more severe pathological phenotype in one complex over another.

Mitoribosome pausing may also reflect a physiological mechanism that coordinates co-translation of interacting polypeptides from the same OXPHOS complex. Analogous to the way in which signal recognition particles bind nascent peptides emerging from cytosolic ribosomes to delay translation, features within the mtDNA encoded transcripts may arrest mitoribosomes until the protruding domain of the nascent peptide docks with its cognate partners. This mechanism could facilitate co-translation both of mitochondrially encoded proteins as well as coordinating intra-mitochondrial translation with the import, or indeed co-translational translocation, of nuclear encoded components of OXPHOS complexes. There are an increasing number of proteins know to be involved in mitochondrial gene expression and complex assembly. Examining the protected RNA profiles from cell lines with mutations in these proteins, as well as in cells depleted of relevant chaperones, insertases or complex assembly factors could help define the level of control that is exerted by the mitoribosome on assembly of the OXPHOS complexes. CRISPR/Cas9 or siRNA technology could also be combined with profiling to target depletion of candidate proteins of known mitochondrial localisation but of unknown function to elucidate the putative roles in translation that these proteins perform, by comparing the depleted profiles against those of controls.

There is further interrogation of data derived from mitoribosome profiling that remains to be done. This will yield both a better understanding of the fundamental mechanisms governing mitoribosome behaviour in mammals, and will also give a molecular insight into the mechanisms causing the specific pathogenic effects associated with different mitochondrial diseases.

Abbreviations: Mitochondrial, mt; transfer RNA, tRNA; mitoribosomal large subunit, mt-LSU; mitoribosomal small subunit, mt-SSU.

## Data availability

Control ribosome profiling data are deposited in NCBI Gene Expression Omnibus (accession codes GSE48933). Raw data from experimental samples is deposited in EBI ArrayExpress ( E-MTAB-6284).

## Supplementary material

**Figure S1. Unsupervised hierarchical clustering of fractional abundance profiles and mitoribosomal profile on *MTCO1***

**A**. Dendrogram showing a hierarchy of similarity between profiles of the fractional abundance of t-RNA footprints across codons for three replicate wild-type control cell lines (C1, 2, 3), three replicate experimental mutant cybrid lines (M 1, 2, 3) and general fractional abundance of codons in the coding regions of mtDNA (mtDNA). The similarity between two profiles, i and j is defined as 1.0 - Cij where Cij is the Pearson's correlation coefficient between profile i and profile j. **B**. Matrix showing Cij for all combinations of i and j. Profiles and correlations enclosed by black lines highlight how unsupervised hierarchical clustering assigns profiles into groups when told to expect three groups. **C**. The mitoribosome distribution across *MTCO1* for 3 repeat of the same cell line was plotted as a proportion of the footprints found over all the mt-ORFs in the library (fractional abundance).

**Figure S2. Predicted structure of *RNA7***

An RNAfold prediction of *RNA7* is presented with a box indicating the position of the overlap of the two sequences encoding ND4L and ND4.

**Figure S3. Distribution of mitoribosomal footprints surrounding the 3’ terminus of *MTCO2***

The primary IGV data presents the mitoribosome footprints from the region covering the stop codon and the short 3’UTR of the *MTCO2* transcript prior to the poly(A) tail. The control data is above and mutant data below. The nucleotide positions from the mtDNA reference sequence are in between with the termination site indicated by X.

**Table S1. Characteristics of the first 36 amino acids in selected open reading frames of human mtDNA.**
