# Supplementary figures and images for: Using mitoribosomal profiling to investigate human mitochondrial translation
Source: Wellcome Open Res. 2018 Jan 29;2:116. Originally published 2017 Dec 11. [Version 2] doi: 10.12688/wellcomeopenres.13119.2 (PMC5771143; doi:10.12688/wellcomeopenres.13119.2)

Figure S1

A

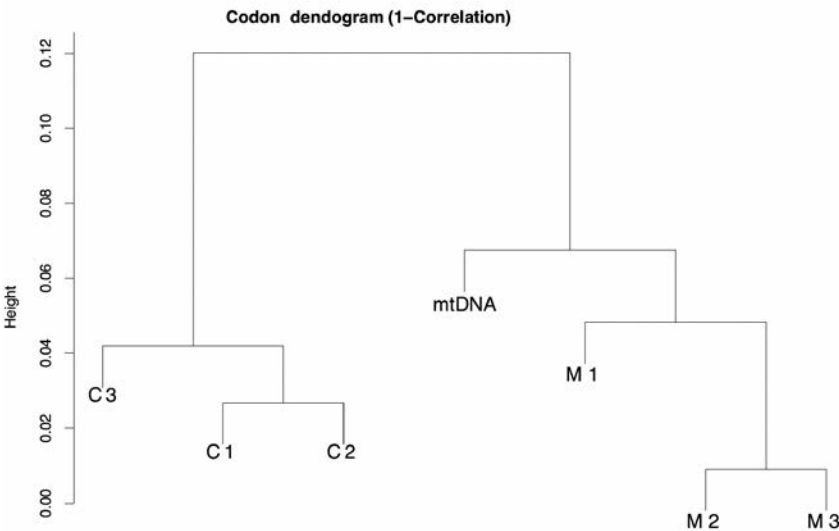

B

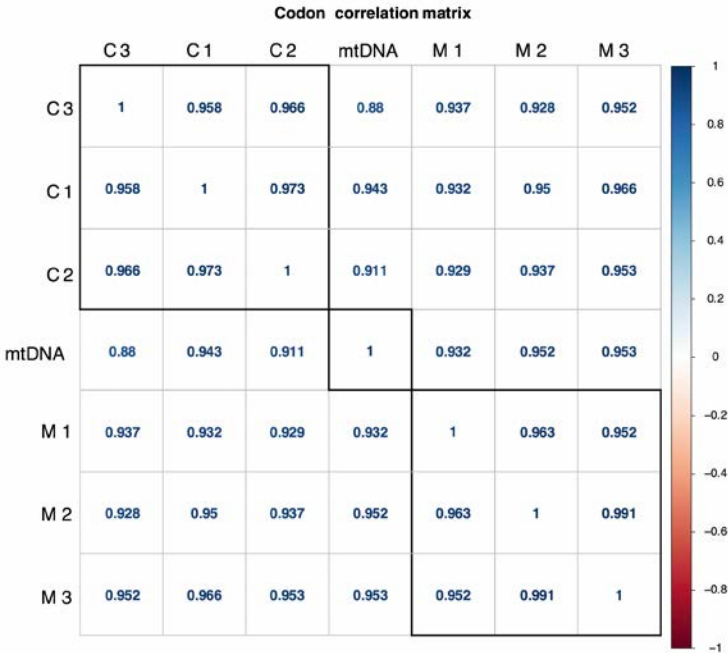

C

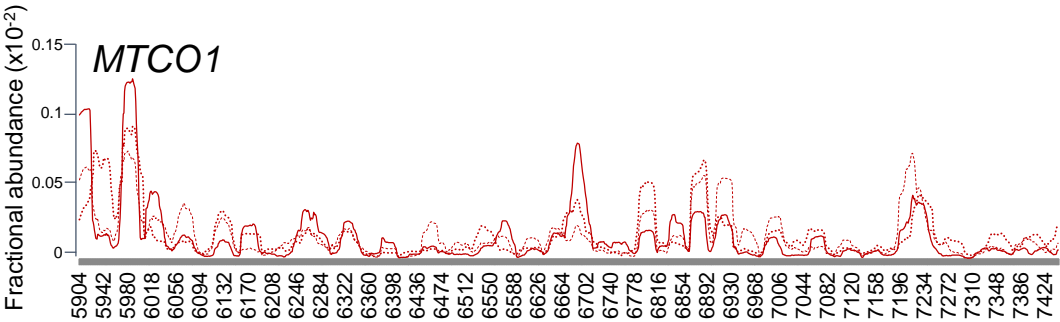

Supplement: Supplementary file 1 [file wellcomeopenres-2-14957-s0004.tgz › 0e67f247-edc6-4eeb-81e0-70ac9f9d9f6b.pdf]

Figure S2

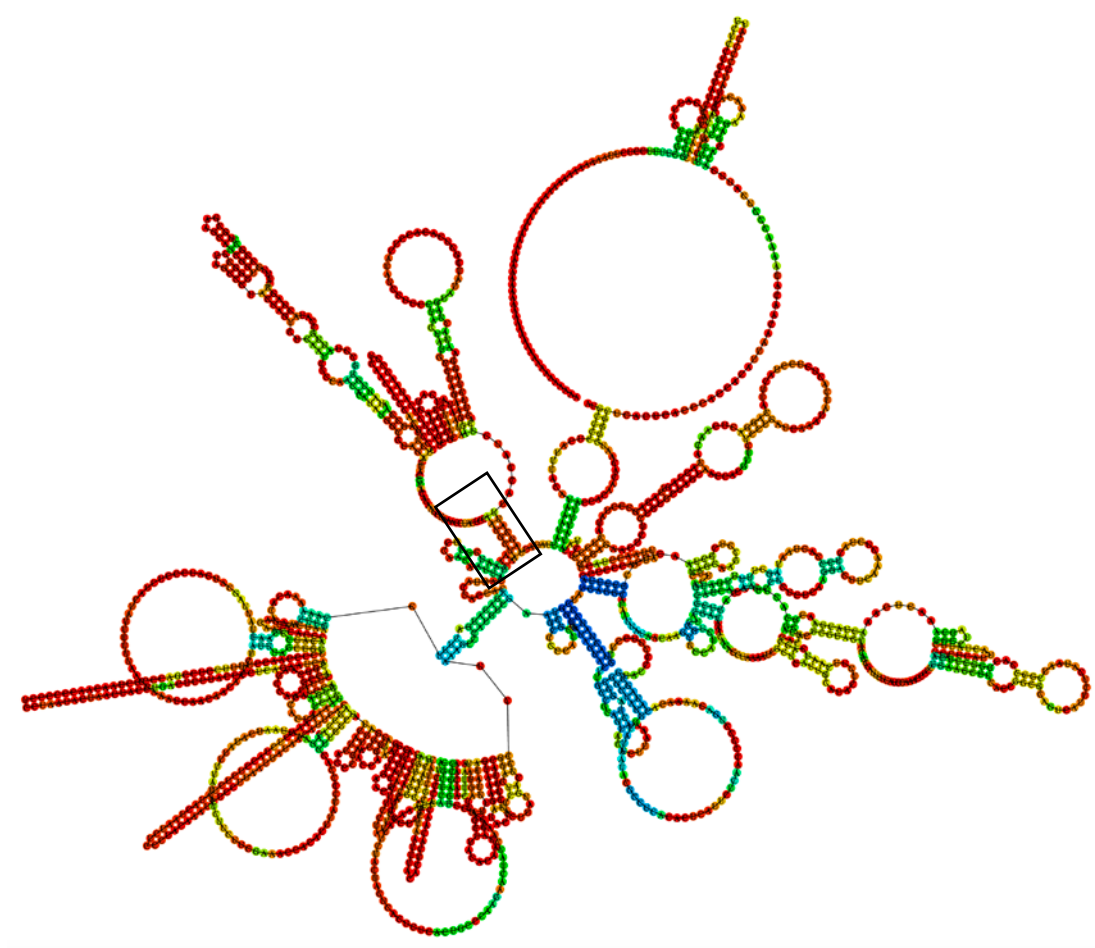

Supplement: Supplementary file 2 [file wellcomeopenres-2-14957-s0005.tgz › 1bffbf94-7ed4-4fc7-be84-16af794b7ee4.pdf]

Figure S3

MTCO2

Control

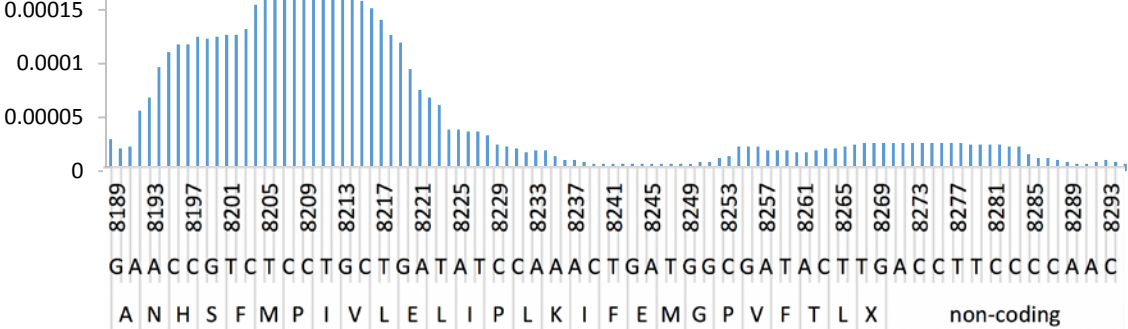

Mutant

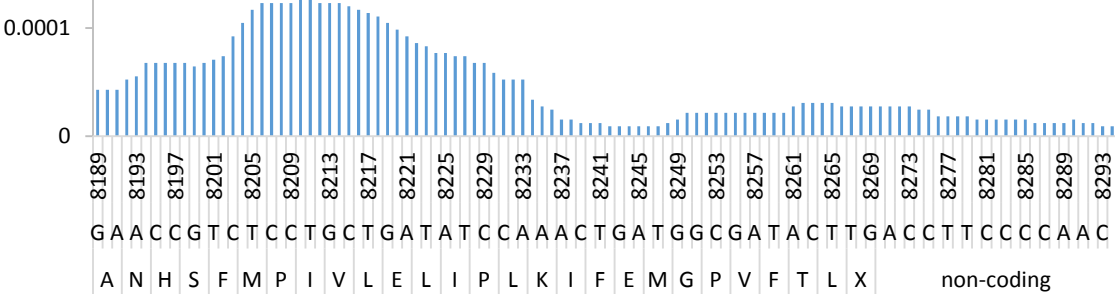

Supplement: Supplementary file 3 [file wellcomeopenres-2-14957-s0006.tgz › 9bb554df-774e-48a9-a9c0-b6c3a4531429.pdf]
