# Supplementary material for: Using mitoribosomal profiling to investigate human mitochondrial translation
Source: Wellcome Open Res. 2018 Jan 29;2:116. Originally published 2017 Dec 11. [Version 2] doi: 10.12688/wellcomeopenres.13119.2 (PMC5771143; doi:10.12688/wellcomeopenres.13119.2)
Supplement: Supplementary file 4 [file wellcomeopenres-2-14957-s0003.tgz › 57bbfd4a-7a54-473a-9380-40d35cb9129b.doc]

Table S1	Characteristics of the first 36 amino acids in selected open reading frames of human mtDNA

| Orf | 1^st^ 36 aa  http://www.ibi.vu.nl/programs/yaspinwww/ | Structure of 1^st^ 36 aa  [**alpha-helix / Chou & Fasman**](http://web.expasy.org/protscale/pscale/alpha-helixFasman.html), | hydrophobicity GRAVY of 1^st^ 36 aa  positive = hydrophobic http://web.expasy.org/protparam/ |
| --- | --- | --- | --- |
| ND2 | [M](http://www.mitomap.org/aaND2.html#AUUfootnote)NPLAQPVIYSTIFAGTLITALSSHWFFTWVGLEMN  AA: MNPLAQPVIYSTIFAGTLITALSSHWFFTWVGLEMN  Pred: --HHHHHHHHHHHHHHHHHHHH-HHHHHHHHHH---  Overall: 994001212101000000110023379985112139  Helix: 009565867647516133472019999999979400  Strand: 000033132352481056527900000000000000  Coil: 990300000000001710000080000000020599 |   0.92-1.12 | 0.700 |
| ND3 | MNFALILMINTLLALLLMIITFWLPQLNGYMEKSTP  AA: MNFALILMINTLLALLLMIITFWLPQLNGYMEKSTP  Pred: --EEEEEHHHHHHHHHHHHHHHHHHHH---------  Overall: 933400001558999899765100124420010179  Helix: 000001199999999999999974789001123000  Strand: 019998800000000000000000000003200000  Coil: 980000000000000000000025210985666999 |   0.9-1.25 | 1.081 |
| ND4L | MPLIYMNIMLAFTISLLGMLVYRSHLMSSLLCLEGM  AA: MPLIYMNIMLAFTISLLGMLVYRSHLMSSLLCLEGM  Pred: --EEEEEEEEEEEHHHHHHHHHHHHHHHHHHHH---  Overall: 953310100000006455587725756766544169  Helix: 000002002666599999999999999999999200  Strand: 009997997333300000000000000000000000  Coil: 990000000000000000000000000000000799 |   0.96–1.16 | 1.300 |
| ND4 | MLKLIVPTIMLLPLTWLSKKHMIWINTTTHSLIISI  AA: MLKLIVPTIMLLPLTWLSKKHMIWINTTTHSLIISI  Pred: -EEEEEEEEEEE-HHHH----EEEEE----EEEEE-  Overall: 830131102610001021685666134200156459  Helix: 000000000000057783000000000000000000  Strand: 089999689998421200000999980046999990  Coil: 910000310001410005999000019853000009 |   0.9-1.15 | 0.986 |
|  |  |  |  |
| COX1 | MFADRWLFSTNHKDIGTLYLLFGAWAGVLGTALSLL    AA: MFADRWLFSTNHKDIGTLYLLFGAWAGVLGTALSLL  Pred: -EEEEEEEE-------EEEEEE-HHHHHHHHHHHH-  Overall: 920201311264422114674200116447430029  Helix: 000000000000000000000027999999999580  Strand: 075679997100000399999900000000000000  Coil: 924320002899999600000072000000000419 |   0.85-1.15 | 0.725 |
| COX2 | MAHAAQVGLQDATSPIMEELITFHDHALMIIFLICF     AA: MAHAAQVGLQDATSPIMEELITFHDHALMIIFLICF  Pred: --------------HHHHHHHHH---EEEEEEEE--  Overall: 986110151000512767984024853157886319  Helix: 000602103445049999999970000000000000  Strand: 000023100000000000000000009999999960  Coil: 999374796544950000000029990000000039 |   0.9-1.2 | 0.908 |
|  |  |  |  |
| ATP6 | MNENLFASFIAPTILGLPAAVLIILFPPLLIPTSKY     AA: MNENLFASFIAPTILGLPAAVLIILFPPLLIPTSKY  Pred: ------------------EEEEEEEE----------  Overall: 990100101241000152326786135310147789  Helix: 009320022003223100000000000000000000  Stand: 000022100100241101999999990044400000  Coil: 990656766895434798000000009955599999 |   0.85-1.15 | 1.214 |
| ATP8 | MPQLNTTVWPTMITPMLLTLFLITQLKMLNTNYHLP  PSPKP     AA: MPQLNTTVWPTMITPMLLTLFLITQLKMLNTNYHLP  Pred: --------------HHHHHHHHHHHHHHH-------  Overall: 987773102000012768547689656132662269  Helix: 000000000754009999999999999990000000  Strand: 000002562023500000000000000000000000  Coil: 999997437222380000000000000009999999 |   0.85–1.2 | 0.442 |
