# Supplementary material for: Using mitoribosomal profiling to investigate human mitochondrial translation
Source: Wellcome Open Res. 2018 Jan 29;2:116. Originally published 2017 Dec 11. [Version 2] doi: 10.12688/wellcomeopenres.13119.2 (PMC5771143; doi:10.12688/wellcomeopenres.13119.2)
Supplement: Peer review history

# Peer review: Referee response for version 1

**DOI:** 10.21956/wellcomeopenres.14231.r29504

**Published:** 2018-01-15

**Authors:** Henna Tyynismaa

**Affiliations:** 1 Research Programs Unit, Molecular Neurology Research Program, Department of Medical and Clinical Genetics, University of Helsinki, Helsinki, Finland

This is an interesting article using mitoribosome profiling to investigate why human cells with mt-tRNAPhe instead of the normal mt-tRNAVal in the mitoribosome have subtly impaired mitochondrial protein synthesis. No significant differences in mitoribosome accumulation were identified over any codons, indicating no increased stalling. Ribosome occupancy profiles appeared slightly changed on some RNAs, in particularly the bicistronic RNAs.

 As the authors note only a couple of studies have so far been published using mitoribosome profiling. Therefore no major studies comparing the ribosome profiles in different cell lines have been reported. Fig 1C in this article suggests that HEK293 and 143B cancel cell lines would differ in the ribosome occupancy on MTCO1, especially around 7586. The nucleotide numbering from 7586 to 8266 in the figure is however confusing, as according to the Cambridge Reference Sequence of the Human Mitochondrial DNA MTCO1 is positioned between 5904..7445, whereas MTCO2 is 7586..8269. I was also not sure what the blue marks are next to the codons in Fig 1A. These would be useful to clarify.

I have read this submission. I believe that I have an appropriate level of expertise to confirm that it is of an acceptable scientific standard.

# Peer review: Referee response for version 1

**DOI:** 10.21956/wellcomeopenres.14231.r29271

**Published:** 2018-01-11

**Authors:** Antoni Barrientos

**Affiliations:** 1 Department of Neurology , University of Miami Leonard M. Miller School of Medicine (UMMSM), Miami, FL, USA

This is an excellent paper reporting on the unexpected occurrence of a structural tRNA on the mitochondrial ribosome large subunit. In addition, the tRNA varies among mammals. While the human mtLSU contains tRNA-Val, the porcine contains tRNA-Phe.

 Here, the authors use mitoribosomal profiling to study the implications of substituting one tRNA by the other in human cells. They observe that tRNA-Phe alter translation fitness. However, the authors demonstrate that this effect is not due to stalling on triplets encoding either Val or Phe. The alterations in translation initiation do not seem to be due either to the presence or absence of 5’ leader sequences, except in the two bicistronic RNA units, RNA7 and RNA14 where the initiation sites are internal. 

 The methods are sound and the results, although negative regarding the selectivity of tRNA -Val vs Phe, are very informative concerning basic questions of how mitochondrial protein synthesis proceeds.

I have read this submission. I believe that I have an appropriate level of expertise to confirm that it is of an acceptable scientific standard.
